# The Financial Condition of the North-Eastern Hospital for Children

**Published:** 1895-11-23

**Authors:** Fredk. Fitz-Roy, Stuart Knill, G. F. Stepney

**Affiliations:** Chairman of Committee.; Alderman, Vice-President.; Bishop Suffragan for East London.


### EDITOR'S LETTER-BOX.

[Our correspondents are reminded that prolixity is a great bar to publication, and that brevity of style and conciseness of statement greatly facilitate early insertion.]

### THE FINANCIAL CONDITION OF THE NORTH-EASTERN HOSPITAL FOR CHILDREN.

Sir,—We ask your help in making known the precarious financial condition of the North-Eastern Hospital for Children, Hackney Road, Shoreditch, an institution that provides treatment in sickness and accident to 15,000 children of the poor annually, and that has worked in this manner for 29 years.

Owing to insufficiency of income in past years a debt of £3,500 (on mortgage at 4 per cent.) has accumulated.

The committee are doing their utmost to prevent further indebtedness, and are striving to pay off the debt and to add £500 to the annual subscription list; but the present necessities of the hospital are so urgent that unless £1,200 can be obtained before January the only alternative to a most disheartening increase of the mortgage will be a restriction of the number of patients.

Sought after and depended upon by thousands upon thousands of poor mothers in the North and East of London, this charity will not surely be allowed to languish for so small a sum.

We appeal to everyone of your readers to give some amount, no matter how small.

Contributions should be sent to the Secretary at the hospital, and cheques should be crossed Barclay, Bevan, and Co.—We are, Sir, your obedient servants,

(Signed)

Fredk. Fitz-Roy, Chairman of Committee.
Stuart Knill, Alderman, Vice-President.
G. F. Stepney, Bishop Suffragan for East London.

